# Combination Therapy With Proton Pump Inhibitors and Iron Chelators in Hereditary Anaemias With Secondary Iron Overload

**DOI:** 10.1002/jha2.70429

**Published:** 2026-10-22

**Authors:** Thomas Doeven, Annelies van Vuren, Andreas Glenthøj, Jean Louis Kerkhoffs, Saskia E. M. Schols, Anita Rijneveld, Erfan Nur, Dore Peereboom, Paco Welsing, Roger Schutgens, Bart J. Biemond, Eduard J. van Beers

**Affiliations:** ^1^ Centre for Benign Haematology Thrombosis and Haemostasis ‐ Van Creveldkliniek University Medical Centre Utrecht Utrecht University Utrecht The Netherlands; ^2^ Department of Internal Medicine Ziekenhuisgroep Twente Almelo The Netherlands; ^3^ Danish Red Blood Cell Centre Department of Haematology Copenhagen University Hospital ‐ Rigshospitalet Copenhagen Denmark; ^4^ Department of Clinical Medicine Faculty of Health and Medical Sciences University of Copenhagen Copenhagen Denmark; ^5^ Department of Hematology HagaZiekenhuis The Hague The Netherlands; ^6^ Department of Hematology Radboud University Medical Center Nijmegen The Netherlands; ^7^ Department of Hematology Erasmus Medical Center Cancer Institute University Medical Center Rotterdam The Netherlands; ^8^ Department of Hematology Amsterdam University Medical Center University of Amsterdam Amsterdam The Netherlands; ^9^ Department of Blood Cell Research Sanquin Research Amsterdam The Netherlands; ^10^ Contactgroep Pyruvaatkinasedeficiëntie Stichting Zeldzame Bloedziekten Medemblik The Netherlands; ^11^ Division of Internal Medicine and Dermatology University Medical Center Utrecht Utrecht University Utrecht The Netherlands

**Keywords:** clinical trial, hereditary anaemia, iron overload, proton pump inhibitors, iron chelation therapy

1

To the Editor:

Secondary iron overload is an important complication of hereditary anaemias. In patients who are not regularly transfused, iron loading is largely driven by increased intestinal uptake resulting from hepcidin suppression [1, 2, 3]. Iron chelation therapy remains the cornerstone of treatment as other therapeutic options are limited, making the intestinal absorption pathway an attractive target [2].

Proton pump inhibitors (PPIs) reduce gastric acid secretion, thereby impairing non‐haem iron absorption by limiting ferric iron solubilization and its reduction to the absorbable ferrous form [4, 5]. Several studies suggest that treatment with PPIs reduces ferritin levels and phlebotomy requirements in *HFE*‐related hereditary hemochromatosis [5, 6, 7, 8]. A subsequent randomized controlled trial in thalassaemia patients showed reductions in ferritin levels in those patients receiving PPIs and iron chelators, whereas no changes were observed in those receiving iron chelation monotherapy, suggesting a potential additive effect [9].

Recently, we have conducted a placebo‐controlled cross‐over clinical trial, evaluating PPI treatment in a heterogeneous cohort of patients with various non‐transfusion‐dependent hereditary anaemias and mild‐to‐moderate iron overload [10]. Patients (*n* = 30) received esomeprazole 40 mg and placebo bidaily for 12 months each. Esomeprazole treatment resulted in a reduction from baseline of 0.57 mg Fe/g dry weight (dw) in liver iron concentration (LIC), as measured by MRI, compared to a reduction of 0.11 mg Fe/g dw in the placebo arm, *p* = 0.033. A pre‐planned subgroup analysis in participants with a low hepcidin/ferritin ratio (below median) showed a reduction from baseline of 1.20 mg Fe/g dw in the esomeprazole arm compared to an increase of 0.08 mg Fe/g dw in the placebo arm, *p* = 0.003, while in participants with high hepcidin/ferritin ratio (above median), LIC reductions were comparable between both arms. Results were similar when stratifying by hepcidin alone, and inflammatory markers were normal [11]. These results suggest that esomeprazole treatment reduces iron overload, and although based on small sample sizes, this effect appeared most pronounced in patients with lower hepcidin levels.

Moris et al. hypothesized that hepcidin suppression occurs due to lower iron availability in the small intestine, induced by PPIs. This hepcidin suppression subsequently increases iron absorption, negating the effects of PPI treatment [12]. However, in patients with already substantially suppressed hepcidin levels, hepcidin might not be suppressed any further. In those patients, the effects of PPI treatment cannot be countered, leading to a net reduction in intestinal iron uptake [12, 13]. This hypothesis might explain the greater LIC reductions observed in patients with low baseline hepcidin. However, we hypothesize that an alternative explanation may also contribute to the observed differences. Given previous evidence indicating that PPIs may enhance the effects of iron chelators in patients with thalassemia, we hypothesize that these therapies may have a potential additive effect on reducing iron overload [9]. Apart from the aforementioned study, the combined effects of PPIs and iron chelation therapy have, to our knowledge, not been investigated before.

This re‐analysis was performed to explore whether this hypothesis is plausible, either as an alternative or as a concurrent explanation for the observed reduction in iron overload. We stratified patients based on iron chelation therapy. All patients on iron chelation therapy were receiving deferasirox film‐coated tablets, and no dose alterations were allowed. Median dose was 5.4 mg/kg/day (IQR 3.9–6.7) over a 12‐month period. One participant lacked dosage information, and one participant used deferasirox for only four months in each treatment period. Adherence was not recorded and, therefore, actual treatment exposure could not be assessed. Patients receiving deferasirox had higher transferrin saturation levels and lower hepcidin levels (Table 1). Importantly, most patients (9 of 11) receiving deferasirox were included in the low hepcidin group in the previously reported pre‐planned subgroup analysis, limiting the ability to determine whether the LIC reduction was attributable to hepcidin levels, combined treatment, or a combination of both.

**TABLE 1:** Baseline characteristics by iron chelation usage.

	**Iron chelation therapy (*n* = 11, 37%)**	**No iron chelation therapy (*n* = 19, 63%)**	***p*‐value** ^a^
Age in years, median (range)	46 (23–56)	47 (19–66)	0.10
Female sex, *n* (%)	4 (36)	11 (58)	0.26
*Diagnosis, n (%)*			—
CSA	3 (27)	—	
CDA	1 (9)	2 (11)	
HE	—	1 (5)	
NTDT	5 (45)	8 (42)	
PKD	2 (18)	6 (32)	
SCD	—	2 (11)	
Prior splenectomy, *n* (%)	3 (27)	4 (21)	1
Prior cholecystectomy, *n* (%)	3 (27)	7 (37)	0.70
Number of blood transfusions in preceding 12 months, median (range)	0 (0–9)	0 (0–4)	0.37
Number of phlebotomies in preceding 12 months, median (range)	0	0	—
*Markers of iron metabolism, median (IQR)*			
Ferritin in µg/L	361 (589)	540 (368)	0.52
Transferrin saturation in %	74.5 (43.8)	45.0 (42.3)	**0.041**
Plasma hepcidin in µg/L	4.2 (6.1)	11.5 (15.0)	**0.005**
Haemoglobin in g/dL, median (IQR)	8.9 (0.8)	9.8 (2.4)	0.14
Baseline LIC in mg Fe/g dry weight, median (IQR)	5.2 (4.7–10.1)	4.6 (3.0–5.8)	0.094

Abbreviations: CDA, congenital dyserythropoietic anaemia; CSA, congenital sideroblastic anaemia; HE, hereditary elliptocytosis; IQR, interquartile range; LIC, liver iron concentration; NTDT, non‐transfusion‐dependent thalassemia; PKD, pyruvate kinase deficiency; SCD, sickle cell disease.

^a^ Using independent *t*‐test or Mann–Whitney *U* test for continuous variables and either chi‐square test or Fisher's exact test for categorical variables.

Bold values indicate statstical significance (*p* < 0.05).

We explored the primary and key secondary endpoints between the two groups. In the intention‐to‐treat analysis of patients receiving deferasirox, treatment with esomeprazole led to a decrease of 1.52 (SD 1.31) mg Fe/g dw in LIC compared to a decrease of 0.22 (SD 0.82) mg Fe/g dw in the placebo group (mean difference: −1.31 mg Fe/g dw [95% CI −2.16 to −0.45], *p* = 0.007) (Figure 1, Table S1). The median deferasirox dose was 4.8 mg/kg/day (IQR 3.2–5.7) during esomeprazole treatment and 5.1 mg/kg/day (IQR 3.2–6.2) during placebo treatment. In contrast, in patients not receiving deferasirox, no significant difference was observed between groups (mean difference: −0.19 mg Fe/g dw [95% CI −0.58 to 0.54], *p* = 0.945). Similar effect sizes were observed in the per‐protocol analysis, although the small subgroups in these analyses limit the robustness of our findings.

**FIGURE 1:**
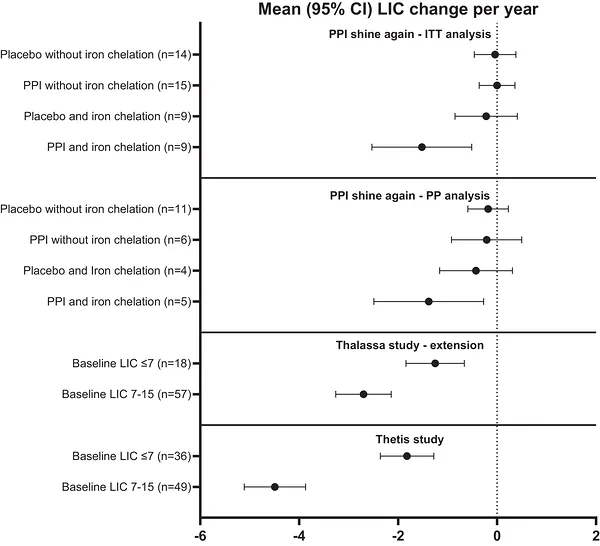
Annual change in liver iron content across studies stratified by subgroups. CI, confidence interval; ITT, intention‐to‐treat; LIC, liver iron content; PP, per‐protocol; PPI, proton pump inhibitor.

As baseline hepcidin levels differed between groups, a sensitivity analysis was performed. As hepcidin was only measured at the start of the study and not remeasured after cross‐over, hepcidin was added as a non‐time‐varying covariate in the primary mixed model analysis. This sensitivity analysis resulted in comparable results (less than 5% change in effect estimate), and all previously significant effects remained statistically significant. To further confirm our findings observed in the subgroups and to explore whether treatment effect of esomeprazole differed according to chelation status, we conducted a treatment‐by‐chelation interaction analysis by including the interaction term (chelation × esomeprazole) in the primary mixed model analysis. In the intention‐to‐treat analysis, this interaction term showed a statistically significant effect (effect estimate: −1.20 [95% CI, −2.20 to −0.20], *p* = 0.020), suggesting an association between combined deferasirox and esomeprazole treatment and LIC change. A comparable but non‐significant effect was observed in the per‐protocol cohort, likely due to limited statistical power associated with the small sample size (effect estimate: −0.83 [95% CI, −1.79 to −0.14], *p* = 0.085). No differences in change of ferritin levels and transferrin saturation were observed when comparing esomeprazole and placebo treatment in patients receiving deferasirox and patients not receiving deferasirox.

The results of this re‐analysis highlight three important findings. First, after 12 months of treatment with deferasirox alone, no effect was observed on LIC (mean change: −0.22 mg Fe/g dw). Second, addition of esomeprazole to the treatment regimen resulted in a considerable reduction in LIC (mean change: −1.52 mg Fe/g dw). Finally, patients without concomitant deferasirox treatment showed no effect of esomeprazole on LIC (mean change: 0.00 mg Fe/g dw). Although the difference in hepcidin levels between groups limits direct causal interpretation, we show that neither of these treatments alone was associated with a decrease in iron overload, suggesting that their individual effects are limited. Co‐administration, however, led to reduction in iron overload, suggesting a potential additive effect of these treatments in this patient population, supported by our findings from the treatment‐by‐chelation analysis.

The LIC reduction observed in the combined treatment subgroup is comparable with results from prior clinical trials evaluating iron chelation therapy in secondary iron overload having similar iron burden (Figure 1). Our cohort consisted of patients with mild‐to‐moderate iron overload (median baseline LIC: 5.0 mg Fe/g dw [IQR 3.7–6.5]). In subgroups of patients with baseline LIC ≤ 7 in both the THALASSA and the THETIS studies, annual LIC decreased by 2.5 (SD 1.2) mg Fe/g dw and 1.8 (SD 1.6) mg Fe/g dw, respectively, while receiving deferasirox dispersible tablets 10 mg/kg/day [14, 15]. Patients with higher baseline iron overload burden exhibited greater LIC decreases, while also receiving higher dosages of deferasirox (Figure 1). The added value of PPIs might seem limited, but for some patients it can tip the net iron balance in the right direction, as observed in our trial.

A limitation of this re‐analysis is the small sample sizes resulting from stratification, especially in the primary and treatment‐by‐chelation analyses of the per‐protocol cohorts. The limited statistical power of these analyses warrants cautious interpretation of the results. In addition, the heterogeneity of our cohort, consisting of patients with disorders that have distinct pathophysiological backgrounds, limits the interpretation of our results given the known differences in the mechanisms and degree of iron loading between these disorders. However, we argue that a shared mechanism contributes to iron overload across these disorders, while the rarity of some of the disorders warranted our basket study design [1, 16]. Furthermore, as data on adherence to deferasirox were absent, the extent to which treatment adherence may have influenced the outcome cannot be assessed. The findings should further be interpreted with caution, as most patients (9 of 11) using deferasirox were also included in the prior low‐hepcidin‐based subgroup analysis (*n* = 14), receiving a median deferasirox dose of 4.8 mg/kg/day (IQR 3.2–5.7). This re‐analysis, however, explores an alternative or concurring explanation for the larger iron reduction effect observed between subgroups. Whether LIC reduction is driven by the combined therapy or whether it is particularly effective in patients with marked hepcidin suppression remains to be further investigated. Nonetheless, deferasirox alone was not associated with a significant LIC reduction in this small subgroup. We speculate that the increased iron uptake due to suppressed hepcidin in this subgroup offsets the iron reduction by chelation.

This exploratory re‐analysis evaluated the effects of combined therapy with esomeprazole and deferasirox on secondary iron overload in hereditary anaemias, showing substantially greater iron overload reduction during co‐administration compared with either therapy alone. The potential additive effect of these therapies warrants further investigation in larger, prospective, randomized controlled trials.

## Author Contributions

**Thomas Doeven**: formal analysis, writing – original draft preparation, writing – review and editing. **Annelies van Vuren**: conceptualization, formal analysis, funding acquisition, investigation, project administration, writing – review and editing. **Andreas Glenthøj**: supervision, writing – review and editing. **Jean Louis Kerkhoffs**: investigation, writing – review and editing. **Saskia E.M. Schols**: investigation, writing – review and editing. **Anita Rijneveld**: investigation, writing – review and editing. **Erfan Nur**: investigation, writing – review and editing. **Dore Peereboom**: writing – review and editing. **Paco Welsing**: formal analysis, writing – review and editing. **Roger Schutgens**: supervision, writing – review and editing. **Bart J. Biemond**: conceptualization, writing – review and editing. **Eduard J. van Beers**: conceptualization, funding acquisition, investigation, supervision, writing – review and editing.

## Funding

The PPI Shine Again (NL6659) has been sponsored by ZonMw, The Netherlands Organization for Health and Research Development, and by the Innovatiefonds Zorgverzekeraars.

## Ethics Statement

The trial was centrally approved by the ethics committee of the University Medical Centre Utrecht (Utrecht, NL) and approved by the Board of Directors of the participating centres. The trial was conducted according to Good Clinical Practice guidelines, defined by the International Conference of Harmonization.

## Consent

All patients provided written informed consent before trial enrolment.

## Conflicts of Interest

Andreas Glenthøj has received research funding from Agios, Bristol Myers Squibb, Novo Nordisk, and Sanofi and is an advisory board member of Agios, Disc Medicine, Novo Nordisk, Pharmacosmos, and Vertex Pharmaceuticals. Anita Rijneveld has received funding for advisory boards from Vertex pharmaceuticals. Bart J. Biemond has received research funding from Pfizer, BMS, and Novo Nordisk and honoraria for consultancy, invited lectures and podcasts from Novartis, Pfizer, BMS/Celgene, Novo Nordisk and Sanofi. Eduard J. van Beers has received research funding from Agios Pharmaceuticals, Vertex Pharmaceuticals, and Novo Nordisk, and holds roles in the Sickle Cell Outcome Register and ERN‐EuroBloodNet. The other authors declare no conflicts of interest.

## Supporting information




**Supporting File 1**: jha270429‐sup‐0001‐SupMat.docx

## Data Availability

Aggregated data are available upon request.
