# Supplementary material for: Combination Therapy With Proton Pump Inhibitors and Iron Chelators in Hereditary Anaemias With Secondary Iron Overload
Source: EJHaem. 2026 Oct 22;7(5):e70429. doi: 10.1002/jha2.70429 (PMC13690014; doi:10.1002/jha2.70429)
Supplement: Supplementary file 1 [file JHA2-7-e70429-s001.docx]

**Supplementary Table 1.** Mean change in liver iron content (LIC) and markers of iron metabolism in the full cohort and subgroups by iron chelation usage

|  | Esomeprazole  mean (SD) | Placebo  mean (SD) | Effect esomeprazole  estimate (95% CI) | P-value |
| --- | --- | --- | --- | --- |
|  | *Full cohort - ITT analysis* | | | |
| Δ LIC in mg Fe/g dw | -0.57 (1.20),  N = 24 | -0.11 (0.75), N = 23 | -0.55 (-1.06 to -0.05) | **0.033** |
|  | *Full cohort – PP analysis* | | | |
| Δ LIC in mg Fe/g dw | -0.74 (0.96),  N = 11 | -0.24 (0.57), N =15 | -0.50 (-1.00 to 0.01) | 0.052 |
|  | *Patients with iron chelation - ITT analysis* | | | |
| Δ LIC in mg Fe/g dw | - 1.52 (1.31), N = 9 | - 0.22 (0.82), N = 9 | - 1.31 (- 2.16 to - 0.45) | **0.007** |
|  | *Patients with iron chelation – PP analysis* | | | |
| Δ LIC in mg Fe/g dw | - 1.38 (0.90), N = 5 | - 0.43 (0.46), N = 4 | - 1.11 (- 1.95 to – 0.27) | **0.024** |
|  | *Patients without iron chelation - ITT analysis* | | | |
| Δ LIC in mg Fe/g dw | 0.00 (0.66),  N = 15 | - 0.04 (0.73), N = 14 | - 0.02 (- 0.58 to 0.54) | 0.95 |
|  | *Patients without iron chelation - PP analysis* | | | |
| Δ LIC in mg Fe/g dw | - 0.21 (0.68), N = 6 | - 0.18 (0.61), N = 11 | - 0.19 (- 0.85 to 0.47) | 0.52 |
| Abbreviations: SD: Standard deviation, LIC: Liver iron content, ITT: Intention-to-treat, PP: Per-protocol | | | |  |
